# Carcass size, not source or taxon, dictates breeding performance and carcass use in a burying beetle

**DOI:** 10.1098/rsos.241265

**Published:** 2024-10-30

**Authors:** Gen-Chang Hsu, Wei-Jiun Lin, Chi-Heng Hsieh, Yue-Jia Lee, Syuan-Jyun Sun

**Affiliations:** ^1^ Department of Entomology, Cornell University, Ithaca, NY, USA; ^2^ Institute of Ecology and Evolutionary Biology, National Taiwan University, Taipei, Taiwan; ^3^ Institute of Food Science and Technology, National Taiwan University, Taipei, Taiwan; ^4^ International Degree Program in Climate Change and Sustainable Development, National Taiwan University, Taipei, Taiwan

**Keywords:** breeding performance, burying beetle, carcass, nutritional composition, offspring quality–quantity trade-off, resource use efficiency

## Abstract

Small vertebrate carcasses represent critical resources for many terrestrial organisms, including burying beetles, which rely on carcasses for survival and breeding. Carcass attributes can influence the reproduction of burying beetles, yet most studies on their breeding ecology have used laboratory-reared carcasses of limited sizes. We conducted breeding and feeding experiments using a wide size range of lab (laboratory mice) and wild carcasses (wild mammals, birds and reptiles) to investigate how carcass size, source and taxon affect various breeding outcomes (e.g. clutch size, brood size and brood mass) of the burying beetle *Nicrophorus nepalensis*. Our results reveal a hump-shaped relationship between carcass size and breeding performance, with optimal breeding outcomes occurring on medium-sized carcasses. Furthermore, despite the variation in carcass tissue nutritional composition, breeding outcomes and larval growth did not differ between the two carcass sources or among the three wild carcass taxa. Finally, we found a larval quality–quantity trade-off across the range of carcasses examined, with carcass size shaping the larval life-history traits. Overall, these results elucidate how carcass resources may influence the breeding performance of burying beetles. Importantly, our study provides solid evidence validating decades of research using lab carcasses to study the reproductive ecology of burying beetles.

## 1. Introduction

Small vertebrate carcasses represent a rich resource for a wide variety of terrestrial organisms, including vertebrate scavengers, saprophagous invertebrates and microbial decomposers [1–3]. These carcass-feeding organisms facilitate the recycling of carcass nutrients and make the resource available to other species [4]. For some species such as burying beetles (*Nicrophorus* spp.), carcasses are particularly important because they serve as not only a food resource but also breeding sites where the offspring grow and develop under parental care [5]. Carcass attributes, therefore, can strongly influence the reproduction of burying beetles.

Carcass size is a key factor for the reproductive success of burying beetles because it determines the amount of resource available for breeding. Brood size and brood mass are generally greater on larger (heavier) carcasses [5–9]. Moreover, parents can adjust their reproductive investment based on carcass size [10]. For example, females lay more eggs on larger carcasses within a certain carcass size range [11], and parents regulate the brood size via filial cannibalism when carcass resource is limited [12]. However, despite the resource benefits, large carcasses can be more difficult to utilize because of greater competition from other carcass-feeding organisms [9], and the energetic costs of processing carcass tissue also increase with carcass size. Such cost–benefit trade-offs suggest that reproductive performance might not necessarily be greater on larger carcasses [13]. A few studies have empirically examined the relationship between optimal carcass size and reproductive output in burying beetles [e.g. 14], yet a general understanding is still lacking.

While most studies on the reproduction of burying beetles have focused on carcass size, the source of carcass may also influence their breeding outcomes. Carcasses in the wild come from animals feeding on diverse diets in various environments. However, most breeding experiments use laboratory mice and chicks, which are usually fed fixed diets and reared in a controlled environment. Consequently, lab and wild carcasses may have considerably different body compositions as well as skin and gut microbiomes [15], and these differences can alter larval survival and growth [1,16]. Therefore, experiments comparing the breeding outcomes of burying beetles on lab versus wild carcasses are essential for evaluating whether the results of past studies are representative of natural patterns. Furthermore, burying beetles have been documented to breed on carcasses from a variety of taxonomic groups [5,17]. Different carcass taxa can vary not only in their abundance but also in their tissue nutritional composition [18], which influence the overall reproductive performance of parents as well as individual larval growth and development [19]. However, it remains unknown how breeding outcomes and larval performance may vary among different groups of wild carcasses.

Studies have shown that brood size and larval mass of burying beetles are often negatively correlated with each other [6,12,20]. Such a trade-off between larval quality and quantity may vary with carcass size [8,21] because resource quantity can shape the life-history traits of organisms [22–24]. However, most studies on the offspring trade-off in burying beetles were conducted under a limited range of carcass sizes, and the results have been mixed because of the differential responses of brood size and larval mass to carcass size [8]. Moreover, carcass sources with different qualities can influence larval performance and thereby alter the trade-off patterns, yet few studies have examined this (but refer to [25]). Therefore, examining breeding outcomes across a wide range of carcass sizes from different carcass sources (e.g. lab and wild carcasses) will help better understand how resource variation affects the offspring life-history trade-off in burying beetles.

In this study, we aimed to understand how various carcass attributes (size, source and taxon) influence the breeding outcomes, larval performance and offspring quality–quantity trade-off in burying beetles. We conducted breeding experiments on the species *Nicrophorus nepalensis*, which has been shown to provide extensive parental care for offspring. First, we examined how breeding outcomes (clutch size, brood size and brood mass) and carcass use efficiency varied across a broad range of carcass size (weight was used as a proxy for size in this study) on lab (laboratory mice) and wild carcasses (wild mammals, birds and reptiles). We further focused on the wild carcasses and compared the larval breeding outcomes and carcass use efficiency of *N. nepalensis* on the three wild carcass taxa. We expected that there would be an optimal carcass size for breeding, and the breeding outcomes may differ between lab and wild carcasses as well as among different wild carcass taxa. We next quantified the tissue nutritional composition of lab and wild carcasses and conducted a larval feeding experiment using carcass tissues from different sources and taxa. We expected that the larvae would perform better when feeding on diets with higher nutritional quality. Finally, we examined the larval quality–quantity trade-off on lab and wild carcasses. We expected a trade-off across a broad range of carcass sizes, and the trade-off pattern would differ between lab and wild carcasses.

## 2. Materials and methods

### 2.1. Breeding experiments

We conducted breeding experiments on *N. nepalensis* from the lab colony established in 2023 [26]. Adult beetles were collected from Taipei and New Taipei City, Taiwan and reared in growth chambers under a relative humidity of 70% and a 10:14 h light:dark cycle. The temperature was set to mimic diurnal temperature fluctuation (mean: 17.8°C; range: 16°C−20°C). This represents the natural temperature conditions during the breeding season (November–April) of *N. nepalensis* in northern Taiwan. A male and female were placed in a plastic breeding container (14.2 cm in diameter and 6.3 cm in height) half-filled with moist commercial potting mix (2 cm in depth, equivalent to 300 ml), and a defrosted carcass was then placed on the soil surface. Frozen dead laboratory mice/rats were used as lab carcasses. Wild carcasses were obtained from the Taiwan Roadkill Observation Network (https://roadkill.tw/eng/home) and the Wild Bird Society of Taipei. These wild carcasses weighed from 1.6 to 99.5 g and consisted of small mammals, birds and reptiles. The carcasses used for breeding experiments were animals that had died within the past four months due to traffic collisions and other accidental causes but not poisoning. Upon discovery, these carcasses were immediately transferred to −20°C freezers for preservation. We paired each wild carcass with a lab carcass of a similar weight (measured to the nearest 0.1 g using an electronic analytical balance ATX224R, Shimadzu, Japan) and applied a sibship design where the two males and the two females used in each lab–wild carcass were from the same family line, respectively, to control for parental genotypes (the males and females came from genetically unrelated families). The breeding containers were maintained under the same environmental conditions as those of the lab colony. Five rounds of breeding experiments were conducted from May 2023 to March 2024 (each with a different beetle parent generation), consisting of a total of 121 lab–wild carcass pairs (14, 76 and 31 wild mammal, bird and reptile carcasses, respectively).

We recorded the clutch size of each breeding container at day 4 by counting the number of eggs at the bottom of the container from the outside. This minimized the disturbance to the carcass and parents while providing an accurate estimate of the exact clutch size (*r* = 0.94, *p* < 0.001, *n* = 70 broods; [27]). Eleven days after beetle pairing, we inspected the carcass to record the brood size (number of larvae) and brood mass (total larval weight; measured to the nearest 0.0001 g). We calculated hatching success as brood size divided by clutch size, average larval mass as brood mass divided by brood size and larval density as brood size divided by carcass weight. We also measured the total weight of breeding containers at the beginning and end of the experiments to estimate the amount of carcass tissue used by parents and larvae during the breeding process (larvae were removed from the carcasses). Carcass use efficiency was calculated as the amount of carcass tissue used divided by the initial carcass weight. All the aforementioned breeding outcomes and carcass use were recorded during the first reproductive bout of the breeding pairs.

### 2.2. Nutritional analysis of carcass tissue

To quantify the nutritional composition of lab and wild carcasses, which is essential for understanding how burying beetles use different types of carcasses, we estimated the protein and fat contents of carcass tissue by adopting a proximate analysis approach as described by Al Shareefi & Cotter [28]. We dissected the carcasses by first skinning the animals and retaining the trunks. Trunk tissue was then separated from the bones with a pair of fine tweezers and a scalpel and divided into viscera (all organs inside the peritoneum) and muscles (all visible muscle parts). We next used a meat tenderizer to pound the viscera and muscles evenly and sampled three pieces of visceral and muscle tissue for each carcass for the analysis of nutritional composition. A total of seven lab mice, seven wild mammals, six wild birds and six wild reptiles were dissected and analysed.

For each tissue sample, we dried approximately 100 mg (106 ± 18 mg) of wet tissue in a 40°C oven for 5 days until all water was removed. To determine the fat content, the dried tissue was thoroughly mixed with 100 μl of −20°C acetone and vortexed for 1 min. The mixture was then placed in a −20°C fridge for a 30 min reaction period [29]. After the extraction, the mixture was centrifuged to separate the components, and the acetone was carefully removed. If the acetone appeared turbid after centrifugation, the solvent was discarded and replaced with fresh acetone for further extraction. The process was repeated until the solvent became clear. The residual solvent was then allowed to evaporate at room temperature for 12 h. After the fat removal process, the final product was weighed to determine the protein content, and the fat content was determined by subtracting the protein weight from the dry weight.

### 2.3. Larval feeding experiments

We conducted larval feeding experiments using the remaining dissected carcass tissue from the nutritional composition analysis. We placed *ca* 400 mg (401 ± 21 mg) of carcass tissue into individual plastic containers filled with moist commercial potting mix (soil volume 3.2 cm × 3.2 cm × 2.7 cm). Newly hatched larvae (5 days after female oviposition) were obtained from pairs of breeding beetles (25 families) from the lab colony and one larva was introduced to each container (*n* = 188). After 5 days of feeding, the larval mass at dispersal was recorded, and larval growth was measured as the larval weight gain during the experimental period.

### 2.4. Data analyses

#### 2.4.1. Breeding outcomes and carcass use efficiency

To examine how clutch size, hatching success, brood size, brood mass and carcass use efficiency varied with carcass size on lab and wild carcasses, we fit generalized linear mixed effects models (GLMMs) with each of the aforementioned breeding outcomes as the response, carcass weight and carcass source as well as their interaction as the fixed effects, and lab–wild carcass pair as the random effect. The pronotum widths of the parents and parent generation were included as the covariates in the models. For clutch size and brood size, we used a negative binomial error distribution with a log link function for model fitting to account for data overdispersion; for hatching success, we used a binomial error distribution with a logit link function; for brood mass, we used a Gaussian error distribution; for carcass use efficiency, we used a beta error distribution with a logit link function. Because clutch size and brood size contained many zero values, we additionally included a zero-inflation structure in the models. We determined whether a quadratic curve better described the relationship between each response and carcass weight by comparing the GLMMs fitted with and without a quadratic term for carcass weight via the likelihood ratio test. Results from the quadratic model were reported if the test was significant (*α* = 0.05).

To compare the brood size, brood mass, average larval mass and carcass use efficiency on wild mammal, bird and reptile carcasses, we fit generalized linear models (GLMs) with each of the aforementioned breeding outcomes as the response and wild carcass taxon as the fixed effect. Carcass weight, pronotum widths of the parents and parent generation were included as the covariates in the models. The error distribution and link function for each of the responses were the same as the GLMMs. Because the carcass range was considerably smaller for reptiles (1.6−64.4 g) than for mammals (3.8−94.8 g) and birds (3.2−99.5 g), we restricted the carcass weight range to that of reptiles (≤64.4 g) so that the results were more comparable among the three wild taxa.

#### 2.4.2. Nutritional composition and larval growth

To compare the nutritional composition between the two carcass sources and the three wild carcass taxa, we fit GLMMs with the proportion of protein/fat as the responses, carcass source/taxon and tissue type (viscera versus muscles) as the fix effects and carcass ID as the random effect (a total of four GLMMs). We used a beta error distribution with a logit link function for model fitting in the GLMMs.

To compare the larval growth between the two carcass sources and the three wild carcass taxa, we fit GLMMs with larval weight gain as the response, carcass source/taxon and tissue type as the fix effects and carcass ID and larval family as the random effects (a total of two GLMMs). Larval mass at hatching was included in the models as a covariate. We used a Gaussian error distribution for model fitting in the GLMMs. To further investigate the effect of nutrient content on larval growth on both carcass sources and wild carcasses only, we fit GLMMs with larval weight gain as the response, proportion of protein, fat and tissue type as the fixed effects, and larval family as the random effects (a total of two GLMMs). Larval mass at hatching was included as a covariate. Dead larvae (*n* = 146) were excluded from the analysis (larval survival rate was 22.3% in the 5 day feeding experiments).

#### 2.4.3. Larval quality–quantity trade-off

To evaluate the trade-off between offspring quality and quantity on lab and wild carcasses, we fit a linear model with average larval mass as the response and larval density, carcass source and their interaction as the predictors. A significant negative slope indicates a larval quality–quantity trade-off.

We fit all aforementioned models using the glmmtmb() function in the R ‘glmmTMB’ package [30]. Model assumptions were checked via the quantile residuals generated from the simulateResiduals() function in the R ‘DHARMa’ package [31]. Predictor significance was assessed with the Wald χ^2^ test via the Anova() function (type II sums of squares) in the R ‘car’ package [32]. Post hoc pairwise comparisons among carcass taxa with the Tukey multiplicity adjustment were conducted via the emmeans() function in the R ‘emmeans’ package [33]. All analyses were performed in R v. 4.3.3 [34].

## 3. Results

### 3.1. Breeding outcomes and carcass use efficiency

**Figure 1.**
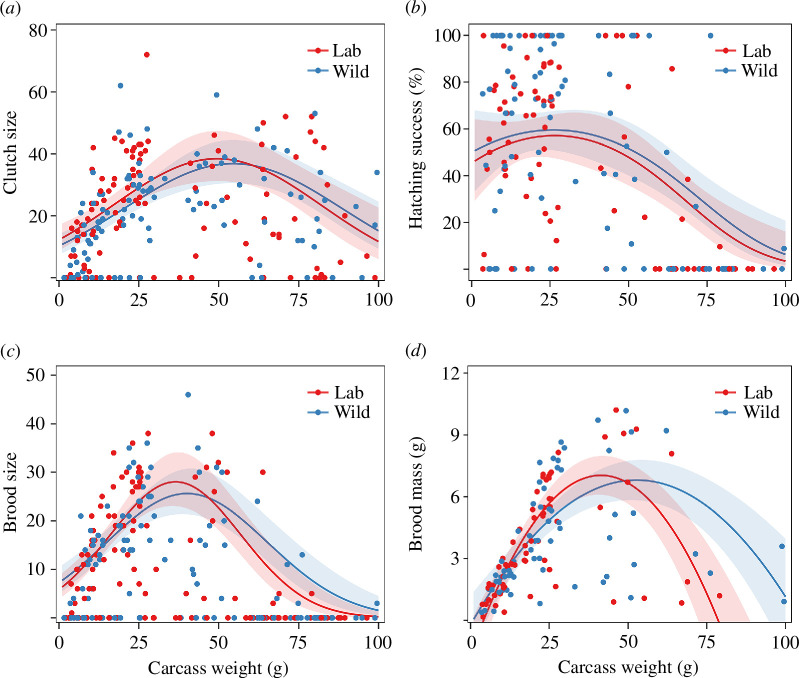
The relationship between carcass weight and clutch size (*a*), hatching success (*b*), brood size (*c*) and brood mass (*d*) on lab and wild carcasses. Note that the observations without any larva were excluded from the brood mass analysis. Lines represent the statistically significant relationships predicted from GLMMs (*α* = 0.05); shaded areas represent the 95% CIs.

**Figure 2.**
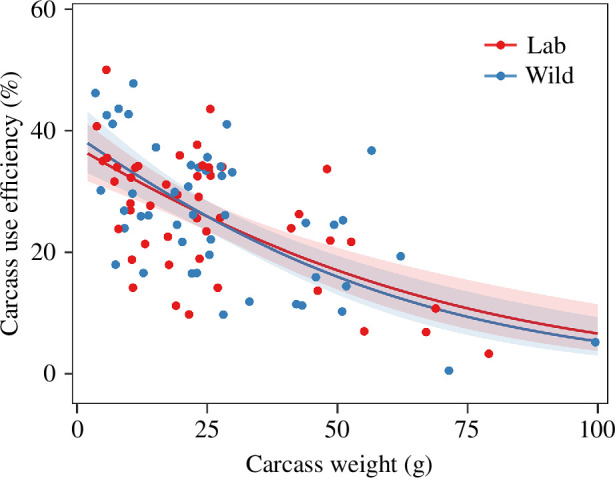
The relationship between carcass weight and carcass use efficiency on lab and wild carcasses. Note that the observations without any larva were excluded from the analysis. Lines represent the statistically significant relationships predicted from GLMMs (*α* = 0.05); shaded areas represent the 95% CIs.

Clutch size, hatching success, brood size and brood mass all showed a quadratic relationship with carcass weight (clutch size: *χ*^2^_2_ = 44.6, *p* < 0.001; hatching success: *χ*^2^_2_ = 32.1, *p* < 0.001; brood size: *χ*^2^_2_ = 63.3, *p* < 0.001; brood mass: *χ*^2^_2_ = 91.9, *p* < 0.001; table 1) and peaked on medium-sized carcasses (figure 1). Moreover, these breeding outcomes did not differ between lab and wild carcasses (clutch size: *χ*^2^_1_ = 1.4, *p* = 0.39; hatching success: *χ*^2^_1_ = 0.8, *p* = 0.37; brood size: *χ*^2^_1_ = 0.009, *p* = 0.93; brood mass: *χ*^2^_1_ = 0.001, *p* = 0.99; table 1; figure 1). Carcass use efficiency decreased with carcass weight (*χ*^2^_2_ = 64.5, *p* < 0.001) but did not differ between lab and wild carcasses (*χ*^2^_1_ = 0.003, *p* = 0.96; table 1; figure 2).

**Table 1.** A summary of the GLMM results for the breeding outcomes and carcass use efficiency of *Nicrophorus nepalensis*. The pronotum widths of the parents and parent generation were included as the covariates in all models.

model response	*n*	predictor		
		carcass weight	carcass source	weight × source
clutch size	210^a^	*χ*^2^_2_ = 44.6, *p* < 0.001	*χ*^2^_1_ = 1.4, *p* = 0.39	*χ*^2^_2_ = 1.9, *p* = 0.24
hatching success	176^b^	*χ*^2^_2_ = 32.1, *p* < 0.001	*χ*^2^_1_ = 0.8, *p* = 0.37	*χ*^2^_2_ = 0.3, *p* = 0.88
brood size	238	*χ*^2^_2_ = 63.3, *p* < 0.001	*χ*^2^_1_ = 0.009, *p* = 0.93	*χ*^2^_2_ = 3.5, *p* = 0.17
brood mass	129^c^	*χ*^2^_2_ = 91.9, *p* < 0.001	*χ*^2^_1_ = 0.001, *p* = 0.99	*χ*^2^_2_ = 11.0, *p* = 0.004
carcass use efficiency	95^d^	*χ*^2^_1_ = 64.5, *p* < 0.001	*χ*^2^_1_ = 0.003, *p* = 0.96	*χ*^2^_1_ = 0.3, *p* = 0.57

^a^ Clutch size was not recorded in the first round of breeding experiments.

^b^ Observations with a zero clutch size were excluded from the analysis.

^c^ Observations with a zero brood size were excluded from the analysis.

^d^ Carcass use was not measured in the first and second round of the breeding experiments; observations with a zero brood size were excluded from the analysis.

**Figure 3.**
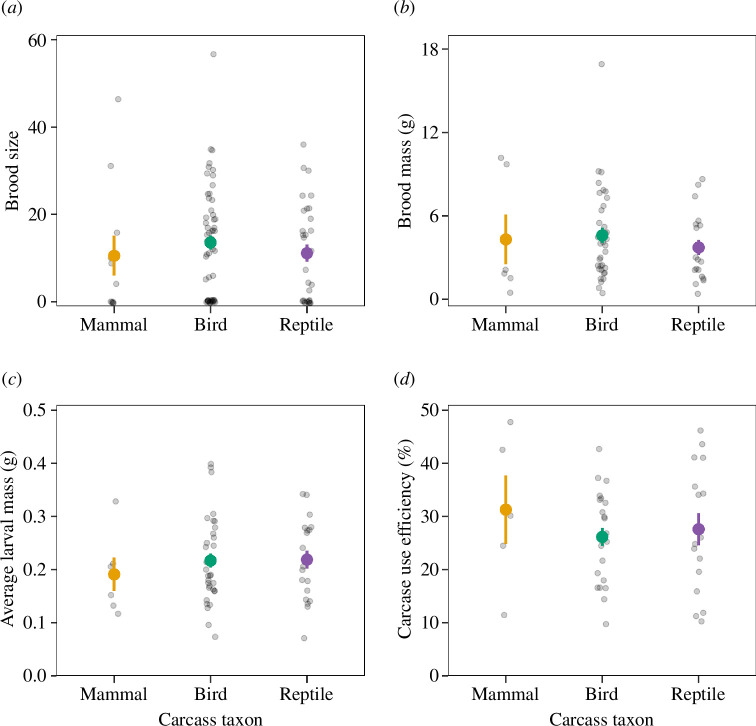
Brood size (*a*), brood mass (*b*), average larval mass (*c*) and carcass use efficiency (*d*) on wild mammal, bird and reptile carcasses. Points represent the means, and error bars represent the standard errors. Note that the observations without any larva were excluded from the brood mass analysis.

Brood size, brood mass, average larval mass and carcass use efficiency did not differ among wild mammal, bird and reptile carcasses (brood size: *χ*^2^_2_ = 0.6, *p* = 0.75; brood mass: *χ*^2^_2_ = 3.6, *p* = 0.17; average larval mass: *χ*^2^_2_ = 3.3, *p* = 0.19; carcass use efficiency: *χ*^2^_2_ = 0.4, *p* = 0.81; figure 3).

### 3.2. Nutritional composition of carcasses

**Figure 4.**
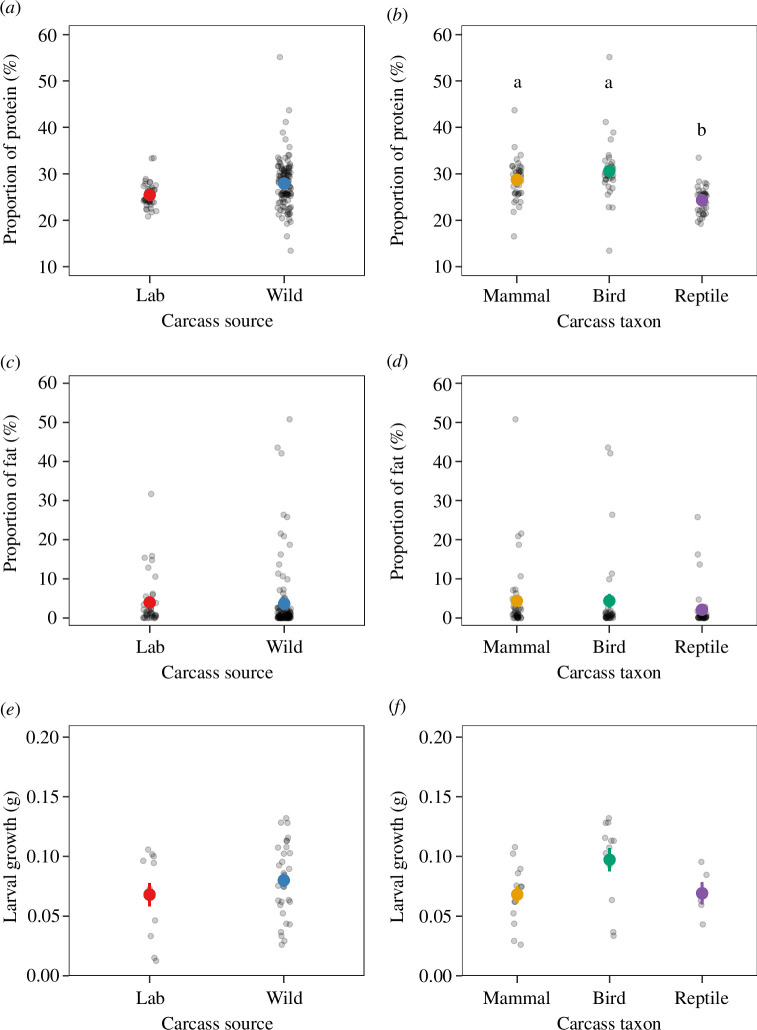
Tissue protein and fat content (*a–d*) and larval growth (*e,f*) on lab and wild carcasses as well as on wild mammal, bird and reptile carcasses. Points represent the means, and error bars represent the standard errors. Letters denote significant difference with Tukey multiplicity adjustment (*α* = 0.05).

Protein content was similar between lab and wild carcasses (mean proportion: lab = 25.5%, wild = 27.9%; *χ*^2^_1_ = 3.5, *p* = 0.06; figure 4*a*) but differed among wild carcass taxa (mean proportion: mammal = 28.7%, bird = 30.6%, reptile = 24.3%; *χ*^2^_2_ = 26.6, *p* < 0.001; figure 4*b*). Specifically, reptile carcasses had significantly lower protein content than mammal and bird carcasses (figure 4*b*). Fat content was similar between lab and wild carcasses (mean proportion: lab = 4.0%, wild = 3.7%; *χ*^2^_1_ = 1.1, *p* = 0.29; figure 4*c*) and among wild carcass taxa (mean proportion: mammal = 4.4%, bird = 4.4%, reptile = 2.1%; *χ*^2^_2_ = 3.5, *p* = 0.18; figure 4*d*).

### 3.3. Larval growth

Growth was similar for larvae feeding on tissue from lab and wild carcasses (*χ*^2^_1_ = 0.1, *p* = 0.74; figure 4*e*). Similarly, larval growth did not differ significantly among the three wild carcass taxa (*χ*^2^_2_ = 5.2, *p* = 0.07; figure 4*f*), although larvae feeding on wild bird carcasses tended to gain more weight compared with those feeding on wild mammals and reptiles (figure 4*f*). When lab and wild carcasses were combined, larval growth was not associated with either tissue protein content (*χ*^2^_1_ = 0.9, *p* = 0.34) or fat content (*χ*^2^_1_ = 0.05, *p* = 0.83) (electronic supplementary material, figure S2a and b). On the other hand, larvae feeding on wild carcass tissue with higher fat content (*χ*^2^_1_ = 5.2, *p* = 0.02), but not protein content (*χ*^2^_1_ = 0.01, *p* = 0.92), did grow better (electronic supplementary material, figure S2c and d).

### 3.4. Larval quality–quantity trade-off

**Figure 5.**
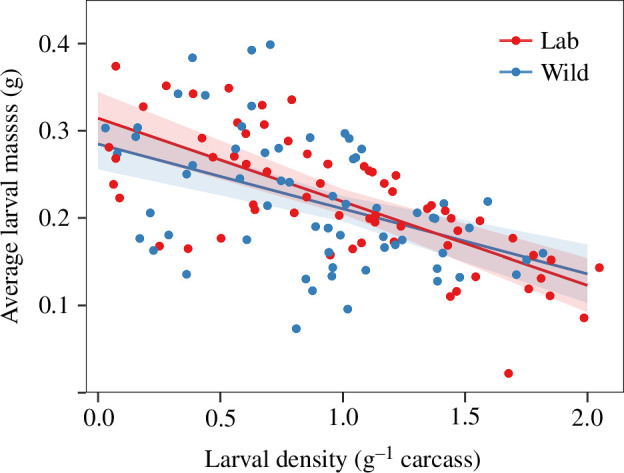
The relationship between larval density and average larval mass on lab and wild carcasses. Lines represent the statistically significant relationships predicted from GLMMs (*α* = 0.05); shaded areas represent the 95% CIs.

Average larval mass decreased with larval density on both lab and wild carcasses (*β* = −0.096, *χ*^2^_1_ = 74.7, *p* < 0.001; figure 5). The interaction between larval density and carcass source was not significant (*χ*^2^_1_ = 1.2, *p* = 0.28), indicating that the trade-off did not differ between lab and wild carcasses (figure 5).

## 4. Discussion

We examined how breeding outcomes and carcass use efficiency of the burying beetle *N. nepalensis* varied with carcass size on lab and wild carcasses. Clutch size, hatching success, brood size and brood mass all exhibited a quadratic relationship with carcass size, whereas carcass use efficiency decreased with carcass size. Furthermore, these breeding outcomes and carcass use efficiency did not differ between lab and wild carcasses. Despite the variation in tissue nutritional composition (protein content) among wild mammal, bird and reptile carcasses, larval traits (brood size, brood mass and average larval mass), carcass use efficiency and larval growth did not differ among the three wild carcass taxa. Finally, a negative relationship existed between larval density and average larval mass on both lab and wild carcasses, suggesting a trade-off between offspring quality and quantity. Taken together, our results indicate that carcass size, but not carcass source or carcass taxon, is the main determinant for the breeding performance and carcass resource use of burying beetles.

As expected, clutch size, hatching success, brood size and brood mass all showed a quadratic relationship with carcass size, with optimal breeding outcomes occurring on medium-sized carcasses. The increase in breeding performance from small to medium carcasses is consistent with previous studies on other burying beetle species [6,10,35]. Interestingly, when the parents bred on large carcasses, their breeding performance decreased, along with a reduction in carcass use efficiency. This may be because large carcasses are more energetically costly to process, and females may lay fewer eggs as a result of lower energy storage. In fact, Müller [11] found that clutch size levels off beyond a certain carcass weight threshold, suggesting an energetic or physiological constraint on beetles breeding on larger carcasses. Parents breeding on large carcasses also face stronger competition with microbes, which can reduce the usable resource for breeding [5] or produce compounds harmful to eggs and larvae [1].

Contrary to our prediction, we found no major difference in the breeding outcomes and carcass use efficiency of *N. nepalensis* on lab versus wild carcasses. A potential explanation is that the parents manipulated the carcasses (e.g. by secreting antimicrobial compounds) such that the eggs and larvae experienced similar growing environments regardless of carcass source. Studies have shown that parental care is crucial for larval performance in burying beetles [1,36], and we speculate that parental food preparation and regurgitation may offset the difference between the two carcass sources. Further experiments comparing breeding outcomes on lab and wild carcasses with versus without parents will help verify our speculation. The analyses did reveal an interaction between carcass size and carcass source for brood mass. In fact, the patterns were mostly similar between lab and wild carcasses on small and medium carcasses, whereas the difference on large carcasses was mainly driven by two observations on large wild carcasses (the interaction became non-significant when these two observations were removed; *p* = 0.38, electronic supplementary material, figure S3). Overall, our results support the validity of research using lab-reared organisms as breeding carcasses to study the reproductive biology of burying beetles.

Our tissue nutritional analysis showed that protein content was higher in wild mammal and bird carcasses than in wild reptile carcasses, whereas fat content was similar among these taxa. Yet, despite the variation in tissue protein content, larval traits and carcass use efficiency in the breeding experiments as well as larval growth in the feeding experiments did not vary significantly among the three wild carcass taxa. In fact, our feeding experiments showed that it was fat content, not protein content, that affected larval growth on wild carcasses. Since fat content did not vary among the three wild carcass taxa, we did not observe major difference in larval growth. This may also partially explain why larval traits and carcass use efficiency were similar among the three wild carcass taxa in the breeding experiments. These results suggest that parents can utilize carcass resources from different vertebrate taxa that vary in their nutritional content and potentially carcass abundance in the wild while having similar breeding outcomes. Without parents, larval survival can be quite low (22.3% in the feeding experiments), and carcass taxon may potentially influence individual larval performance, as larvae did tend to grow better on bird carcasses in the feeding experiments. This highlights the importance of parental care in burying beetles (e.g. carcass preparation and food provisioning) in maintaining breeding performance on a variety of carcasses in the wild.

The negative relationship between average larval mass and larval density on both lab and wild carcasses indicates a trade-off between offspring quality and quantity regardless of carcass source. Similar trade-off patterns have been shown in previous studies [21,37] and can arise from both larval competition and brood regulation by parents [37]. Stronger interspecific competition under a higher larval density may reduce individual larval growth, leading to lower average larval mass. On the other hand, parents may regulate brood size by culling excess larvae to reduce larval competition [38], thereby leading to greater larval growth and higher average biomass. Furthermore, the slope of the negative relationship between average larval mass and larval density did not depend on carcass source, agreeing with our findings that brood size and brood mass did not differ between lab and wild carcasses. Interestingly, we found that the average larval mass increased with carcass size for small and medium carcasses, whereas larval density decreased (electronic supplementary material, figure S1). This suggests that the larval life-history traits of burying beetles can shift depending on breeding resource availability, with smaller carcasses favouring larval quantity (per capita carcass resource) and larger carcasses favouring larval quality.

Our results illustrate the role of carcass size in the breeding outcomes of a single parent pair. However, multiple males and females may engage in cooperative breeding to better utilize large carcasses in the wild [39], although past results for the reproductive benefits of cooperation are mixed [40–42]. Additionally, burying beetles in nature may face carcass competition not only from microbes but also from various vertebrate scavengers and invertebrate carcass feeders [43,44], and such interspecific competition can interact with carcass size to influence breeding success [45]. Abiotic factors such as temperature may further affect the optimal carcass size by altering carcass decomposition rates, carcass handling time and parents’ activity levels [46]. Therefore, field experiments using a wide range of carcass sizes will help elucidate how intraspecific and interspecific interactions as well as the interplay between biotic interactions and carcass size jointly shape the breeding performance of burying beetles. It is also noteworthy that burying beetles can reproduce multiple times throughout their lifespans, and the patterns of single reproductive output may differ from the lifetime reproductive output [14]. Research quantifying the lifetime reproductive output will help better understand how carcass resources affect the over fitness of breeding individuals.

Using a broad range of carcass sizes from both lab and wild sources, our study revealed a quadratic relationship between breeding performance and carcass size in burying beetle, with optimal breeding outcomes occurring on medium-sized carcasses. Breeding outcomes did not differ between lab and wild carcasses. Furthermore, despite the variation in tissue nutritional composition (particularly protein content) among wild mammal, bird and reptile carcasses, larval traits, carcass use efficiency and larval growth were generally similar among these wild carcass taxa. Finally, the larval quality–quantity trade-off existed across the range of lab and wild carcass sizes, with higher larval quantity (larval density) but lower quality (average larval mass) on smaller carcasses and lower larval quantity but higher larval quality on larger carcasses. Taken together, our study confirms that previous results from lab carcasses are fairly representative of natural patterns and provides a more complete picture of how carcass resources shape the breeding performance of burying beetles.

## Data Availability

Data and code used in this manuscript are publicly available on Zenodo [47]. Supplementary material is available online [48].
